# Unpacking early risks for peer victimization: A network analysis of early temperament and polygenic risk scores

**DOI:** 10.1002/jcv2.70092

**Published:** 2026-02-01

**Authors:** Tom C.‐H. Wu, Deniz Konac, Liliana Garcia‐Mondragon, Alex F. Martin, Barbara Maughan, Ted Barker

**Affiliations:** ^1^ Department of Psychology Institute of Psychiatry, Psychology & Neuroscience King's College London London UK; ^2^ Division of Psychology and Language Sciences University College London London UK; ^3^ Department of Psychology Faculty of Humanities and Social Sciences Adana Alparslan Turkes Science and Technology University Adana Turkey; ^4^ International Max Planck Research School for Translational Psychiatry Munich Germany; ^5^ Social, Genetic & Developmental Psychiatry Centre Institute of Psychiatry, Psychology and Neuroscience King's College London London UK

**Keywords:** ALSPAC, difficult temperament, network analysis, peer victimisation, polygenic risk scores

## Abstract

**Background:**

Children who show difficult temperament are at risk of peer victimisation, which in turn associates with numerous negative outcomes later in life. We used network analysis to examine whether specific aspects of difficult temperament contributed to these associations, and whether the links were moderated by variations in genetic liability for ADHD, schizophrenia, and depression.

**Methods:**

In 3354 mother‐child dyads (51.8% female), we examined in three steps: (i) the network structure of difficult temperament as indexed by *adaptability*, *intensity*, and *mood* (age 2), (ii) its item‐level associations with peer victimisation (ages 8, 10, and 13), and (iii) moderation of these associations by polygenic risk scores (PRS) for ADHD, schizophrenia, and depression.

**Results:**

Indicators of difficult temperament formed a coherent network that was associated with peer victimisation. Regarding PRS moderation, for those high in PRS (top 10%) for ADHD and schizophrenia, indicators of temperamental *intensity* and *mood* were associated with peer victimisation, respectively. For those high in PRS for depression, however, aspects of temperament were no longer associated with peer victimisation. Finally, the network results for those in the general population across all PRS (bottom 90%) largely resembled the model estimated using the full sample.

**Conclusion:**

The findings highlight specific temperamental behaviours as risk factors for peer victimisation; additionally, children high in PRS for neurodevelopmental disorders may be at especially high‐risk for this outcome early in development.

## Key Points

**What's known?**

- Early difficult temperament is associated with peer victimisation, but which *specific aspects* of difficult temperament portend higher risks remains unclear.

**What's new?**

- Nodes of temperamental *intensity* (‘Screams when frustrated’) and *adaptability* (‘Shows strong reaction to failure’ and ‘Does not accept delay for desired objects’) were found to be the most central aspects of the difficult temperament network;
- The node ‘Screams when frustrated’ was directly associated with peer victimisation.
- Peer victimisation was also associated with nodes of difficult temperament among those with high genetic risks for neurodevelopmental disorders but not for depression.

**What's relevant?**

- Our findings highlight features of temperamental difficulties associated with peer victimisation, and how these associations vary as a function of genetic risks for psychiatric problems.

## INTRODUCTION

Peer victimisation is a prevalent social problem that has significant impacts on victims. An estimated 32% of school students worldwide have reported being victims of repeated, unwanted, and negative actions from their peers at least once in the past month (UNESCO, 2019); this proportion is likely to be even higher in younger children, as peer victimisation tends to decrease with age (Dulmus, et al., 2006; Twardowska‐Staszek et al., 2018). Child and adolescent victims of bullying are more likely to develop depression, anxiety, and suicidality, risks that persist even into middle adulthood (Takizawa et al., 2014). Given its prevalence and lasting impact, early identification of risk factors and prevention targets for childhood peer victimisation is paramount.

### Associations between difficult temperament and peer victimisation

The role of difficult temperament as a risk factor for peer victimisation is well‐established at the construct level using summed risk score approaches (e.g., Atherton et al., 2017; Hanish et al., 2004; Peterson et al., 2018). However, less is known about which specific *aspects* of difficult temperament predispose children to such adversity. To this end, network analysis may be a useful tool. e.g., Rouquette et al. (2018) have estimated network models of psychopathology in childhood, then tested if symptoms important to these networks were associated with future diagnoses of anxiety and depression. Indeed, the 5 most central symptoms, such as ‘irritable’, were consistently associated with future diagnosis of anxiety disorder, providing insights of higher clinical utility with regards to which symptoms portend higher risks for later psychopathology. Using a similar approach, the present study seeks to build on the difficult temperament literature. First, the network structure of difficult temperament will be examined to identify features of temperamental difficulty central to the network. For instance, which aspects of temperament readily activate with other nodes (i.e., high strength and closeness) or link different clusters of symptoms/traits (i.e., high bridge expected influence). Second, we will examine, at the item‐level, if specific temperamental features are associated with risks for peer victimisation.

Thomas and Chess (1977) operationalized difficult child temperament as being indexed by low *adaptability* to the environment, high *intensity* of emotional expression, predominantly negative *mood*, low *approach* to novelty, and reduced biological *rhythmicity*. Temperamentally difficult children with challenges in emotional and behavioural self‐regulation may be at increased risk for peer victimisation because such difficulties could elicit negative appraisals from peers (e.g., Godleski et al., 2015; Hanish et al., 2004). Whereas, to date, few studies have examined difficult temperament as defined by Thomas and Chess in relations to later peer problems, research has nonetheless shown that scale scores of related operationalisations of difficult temperament are associated with later peer victimisation. For instance, higher mother‐ and child‐reported behavioural regulation (similar to *adaptability* in Carey Toddler Temperament Scale) and tendency to experience negative mood (similar to *mood* in Carey Toddler Temperament Scale) in childhood and adolescence have been found to associate with later peer victimisation (Atherton et al., 2017). Rosen et al. (2012) also found that high negative emotion and poor regulation (e.g., damaged things when mad; angry when frustrated) in children were concurrently associated with victimisation experience and at a 6‐month follow‐up, even after controlling for peer victimisation at baseline.

While these associations are well‐documented at the higher‐order level, it is unclear if (and if so, which) specific features of temperamental difficulty portend higher risks for peer victimisation. Using network analysis, we can test not only, for example, whether temperamental adaptability is associated with peer victimisation, but also which specific indicators are associated with the outcome.

### Moderation by polygenic risk scores

The associations between difficult temperament and peer victimisation may be moderated by individual differences in genetic propensity for mental health problems that are associated with peer victimisation and traits indicative of temperamental difficulty. Here, previous research has identified polygenic risk scores (PRS) that associate with peer victimisation—those for neurodevelopmental disorders such as ADHD and schizophrenia, as well as depression (Pergola et al., 2019; Schoeler et al., 2019). These PRS were suggested to be causally involved in the aetiology of experiencing peer victimisation. A potential mechanistic pathway, for which there is good evidence at the phenotypic level, is that these PRS may code for aspects of difficult temperament, which lead to increased vulnerability for peer victimisation. Specifically, PRS for ADHD and schizophrenia have been found to significantly associate with *intensity* and *mood* at age 2, respectively (Riglin et al., 2022), while PRS for depression are associated with negative moods (Kwong et al., 2021; Navrady et al., 2018). The literature thus suggests that children with higher PRS for these mental health disorders may be at elevated risk for peer victimisation. This would be consistent with an evocative gene‐environment correlation, whereby genetically‐influenced phenotypes evoke certain responses from the environment (Jaffee & Price, 2007). Alternatively, in line with active gene‐environment correlation, children may create environments that align with their genetic propensities (Plomin et al., 1977). Here, temperamentally difficult children may get into more trouble at school due to emotional and behavioural dysregulation, and are thus more likely to be surrounded with others who also exhibit higher levels of behavioural problems and who are more likely to be peer victimisation perpetrators (Georgiou & Stavrinides, 2012).

One way to investigate moderation by PRS is to compare the temperament‐victimisation network for individuals high vs. low in PRS for these mental health problems and see if the association between difficult temperament and peer victimisation is stronger among those with elevated genetic risks. To summarise, we examine in this study using a network approach: (i) the network structure of difficult temperament, (ii) how specific indicators of difficult temperament associate with later peer victimisation, and (iii) whether these associations are stronger among children with elevated polygenic risks for neurodevelopmental disorders and depression.

## METHODS

### Sample

The Avon Longitudinal Study of Parents and Children (ALSPAC) is an ongoing birth cohort study investigating determinants (e.g., genetic and environmental) of health and development of children. Female residents in the former Avon Health Authority were eligible if they were pregnant and had an expected delivery date between April 1st, 1991 and December 31st, 1992 (Fraser et al., 2013). The initial cohort consisted of 14,541 pregnant women, with 13,988 children alive at 1 year of age. Compared to the 1991 National Census Data, the initial sample was predominantly typical of the UK general population (Boyd et al., 2013). The present analytic sample consists of mothers and children of primarily White ethnicity (98.6%).

Ethical approval for the study was obtained from the ALSPAC Law and Ethics Committee and the Local Research Ethics Committees. The ALSPAC website features further information on ALSPAC data (http://www.bris.ac.uk/alspac/), as well as a fully searchable data dictionary and variable search tool (http://www.bristol.ac.uk/alspac/researchers/our‐data/).

### Measures

*Difficult temperament* was measured at age 2, using the Carey Toddler Temperament Scale (CTTS; Carey & McDevitt, 1978). Mothers rated their children's temperamental characteristics on a 5‐point scale ranging from 1 (‘almost never’) to 5 (‘almost always’). Three of the five dimensions of difficult temperament were included (Thomas & Chess, 1977), measured with the most internally consistent items: mood (3 items, e.g. ‘is pleasant during face‐washing’), intensity (4 items, e.g. ‘screams when frustrated’), and adaptability (4 items, ‘e.g. accepts delay for desired objects’). Studies have found support for a second‐order factor structure underlying difficult temperament, with first‐order factors of mood, intensity, and adaptability indexed by these items (Wu et al., 2022a, 2022b). All items were coded such that higher score indicates more difficult temperament. All subscales demonstrated acceptable reliability, ranging from *α* = 0.67 (adaptability) to *α* = 0.74 (intensity).

In addition to internal reliability and factor structure, studies have examined the inter‐rater reliability and convergent validity of mother‐reported CTTS ratings. First, maternal ratings at ages 2‐3 have been found to converge with ratings by day‐care caregivers on all 5 difficult temperament dimensions plus activity (Northam et al., 1987). Second, the scale has also demonstrated strong convergent validity (Goldsmith & Rothbart, 1991) when compared with other temperament scales for toddlers such as the Emotionality Activity Sociability Temperament Survey (EAS; Buss & Plomin, 1975), especially between the mood subscale of the CTTS and emotionality subscale of the EAS.

*Peer victimisation* was assessed at ages 8, 10, and 13 years, using a modified version of the Bullying and Friendship Interview Schedule (BFIS; Wolke et al., 2001). At each time point, trained psychology graduates asked the study child about the frequency with which he or she experienced each of 9 different types of peer victimisation in the past 6 months; these include 5 items measuring overt victimisation (e.g., ‘threatened/beaten up’) and 4 items measuring relational victimisation (e.g., ‘Told lies about’). Answers ranged from 0 (‘did not happen’) to 3 (‘At least once per week’). Responses for each item were first summed across all 3 time points to create 9 items that capture the variance across time, which were then used to create an overall factor score to be included in the networks. Using factor scores offers several advantages, such as the measurement being modelled at the latent level and the error component being separated from the shared factor variance (DiStefano, Zhu, & Mîndrilă, 2009). The overall scale (*α* = 0.79), as well as both overt (*α* = 0.74) and relational (*α* = 0.72) victimisation subscales demonstrated good reliability.

Factor scores for this variable were created by conducting a CFA, using the 9 summed responses described above. Here, a bifactor structure consisting of a general peer victimisation factor and two orthogonal specific factors of overt and relational victimisation provided the best fit to the data. Please refer to Appendix S1 and Table S1 for details on model comparison. Three bifactor‐specific indices were examined. First, the general factor demonstrated high degree of model‐based reliability (*ω* = 0.84). Second, a high construct replicability (*H* = 0.80) suggests that this general factor is a well‐defined and replicable latent variable (Hancock & Mueller, 2001). Finally, the omega hierarchical coefficient (*ω*_*H*_) indicated that this general factor accounts for 72.6% of variance in the total score. The factor score of this general peer victimisation variable was log‐transformed to ensure normality and included as a single node in the networks.

### Polygenic risk score (PRS) calculation

We calculated three PRS using PRSice‐221 (Choi & O'Reilly, 2019). Summary statistics from four recent genome‐wide association studies (GWAS), which do not include ALSPAC, were used as the base data: ADHD (Demontis et al., 2019), schizophrenia (Pardiñas et al., 2018), and major depressive disorder (Howard et al., 2019). We obtained single‐nucleotide polymorphisms (SNPs) in linkage equilibrium by setting the clumping parameters to *r* ^2^ > 0.25 over 250 kb sliding windows. We then created the PRS by weighting the effect sizes of the SNPs that were associated with each of the four traits from the initial GWAS, with a *p*‐value threshold of 1. In order to control for population structure, PRS were adjusted for the first 11 genetic principal components. PRS were standardized and higher scores indicates higher genetic liability to each trait.

### Included versus excluded sample

Participants were included in the study if their PRS were available, and if they provided full data on the BFIS and no more than 2 missing responses to the CTTS; this resulted in a sample of 3354 children (51.8% female). Using multivariate logistic regression, we examined if study variables and demographics predicted inclusion/exclusion from the analytic sample. Odds ratios (ORs) indicated that children excluded from the analysis were more likely to (i) be male (OR = 0.87, *p* < 0.001), and (ii) have lower IQ (OR = 0.98, *p* < 0.001). No significant differences were found for scores on difficult temperament, peer victimisation, and PRS.

### Analysis

The analyses were conducted in three main steps. First, we examined the network structure of difficult temperament alone, where adaptability (*n* = 4), intensity (*n* = 4), and mood (*n* = 3) items were specified as three different communities when calculating bridge centrality. Second, we further included the node representing a factor score of peer victimisation. Finally, we estimated and compared networks for participants with high (i.e., top 10%) versus low (i.e., bottom 90%) PRS in ADHD, schizophrenia, and depression. We then tested differences in network structure and global connectivity.

### Network estimation

All analyses were conducted using R (version 3.6.1; R Core Team, 2019). We estimated the networks using an unregularized Gaussian graphical model (GGM; Costantini et al., 2015), which depicts undirected associations among variables (i.e., nodes). Specifically, the ggModSelect function from the package *qgraph* (version 1.6.9; Epskam et al., 2012) was selected, which uses Bayesian information criterion to choose the best GGM that balances parsimony and goodness‐of‐fit. Partial correlations that represent conditional dependence among nodes were calculated based on Spearman's correlation matrix. In other words, any association (i.e., edge) between pairs of nodes identified existed after controlling for all other variables in the network (Schellekens et al., 2020). To note, the networks were not regularized because of the relatively high sample size compared to the dimensionality (*p* < *n*), and because unregularized networks have been suggested to be more effective in identifying the true network model (Williams & Rast, 2020). We used these unregularized GGM to assess the network structure of difficult temperament and how it associates with peer victimisation.

Node centrality was assessed by using the node strength, closeness, and the 1‐step Bridge Expected Influence (BEI). The centrality index of node strength was calculated by taking the absolute sum of all edges a node forms with any node in the network, which is indicative of how strongly this node connects to others (Opsahl et al., 2010). Closeness centrality demonstrates the average distance between any given node and all other nodes in the network (Forbush et al., 2016). The 1‐step Bridge Expected Influence index (BEI), a bridge centrality index, was used to determine how well a node connects with nodes in other communities. Specifically, the BEI was used to determine which specific aspects of difficult temperament acted as bridge nodes that connect different communities. BEI is calculated by taking the sum of edge weights from a node to all other nodes outside of its community and can also be used to determine the node's influence on the overall network activation because it distinguishes between positive and negative edges (Robinaugh et al., 2016). The nodes with top 20% scores are considered to be bridge nodes in the network (Jones et al., 2019). The BEI has also been demonstrated to be the most robust index at high sample sizes when compared to other bridge centrality indices (Jones et al., 2019). The packages *qgraph* (ver. 1.6.9) and *networktools* (ver. 1.3.0; Jones & Jones, 2017) were used for the estimation of centrality indices.

The accuracy of edge weights and stability of the centrality indices were assessed using the R package *bootnet* (ver. 1.4.3; Epskamp et al., 2018). To examine the accuracy of edges, 95% confidence intervals (C.I.) were constructed for each edge weight by performing nonparametric bootstraps (*n* = 2500). Correlation stability coefficients (CS‐coefficient) were also calculated to examine the stability of centrality indices. Here, case‐dropping bootstraps were performed to test what proportion of the sample can be dropped from the analysis but still yield values that correlate strongly with the original estimates (*r* > 0.7). Only parameters with CS‐coefficients above 0.50, a suggested cut‐off score (Epskamp et al., 2018), were reported.

### Network comparison

We tested for differences in global structure and connectivity (i.e., how strongly the edges are connected) of networks that were estimated separately for participants in the top 10% and bottom 90% of each of PRS, which resulted in 6 networks. We used the *NetworkComparisonTest* package (van Borkulo et al., 2017) in R, a 2‐tailed permutation test that repeatedly (100,000 times) calculates differences between 2 subgroups (e.g., top 10% ADHD and bottom 90% ADHD) with randomly regrouped participants. The permutation yields a distribution that allows one to test whether the observed differences between the groups achieve statistical significance (*α* = 0.05).

## RESULTS

### Descriptive statistics

As shown in Table 1, the summed scores of adaptability and intensity, but not mood, were significantly correlated with peer victimisation. Adaptability was marginally correlated with PRS for ADHD (*p* = 0.053). Intensity was correlated with all three PRS. On the other hand, mood was only significantly correlated with PRS for schizophrenia. Peer victimisation was significantly correlated with PRS for ADHD and depression, although these associations were small in effect (*r* = 0.03 to 0.08) Finally, PRS were significantly correlated among one another.

**TABLE 1:** Descriptive statistics and bivariate associations for study variables.

	Adaptability	Intensity	Mood	Victimisation	PRS_ADHD_	PRS_schizophrenia_	PRS_Depression_
Adaptability	1						
Intensity	0.38***	1					
Mood	0.27***	0.30***	1				
Victimisation	0.07**	0.07**	0.03	1			
PRS_ADHD_	0.03	0.03*	0.02	0.08**	1		
PRSs_chizophrenia_	0.03	0.05**	0.05**	0.01	0.05**	1	
PRS_Depression_	0.02	0.05**	0.03	0.06**	0.15***	0.12***	1
M	11.19	13.34	8.54	0.00	0.00	0.00	0.00
SD	2.85	3.01	2.71	0.86	1.00	1.00	1.00
Min	3.00	4.00	3.00	−0.85	−3.578	−3.29	−4.36
Max	20.00	20.00	15.00	5.30	3.33	3.38	3.44

*Note*: Victimisation = peer victimisation; *p < 0.05; **p < 0.01; ***p < 0.001.

### Network structure of difficult temperament at age 2 (step 1)

**FIGURE 1:**
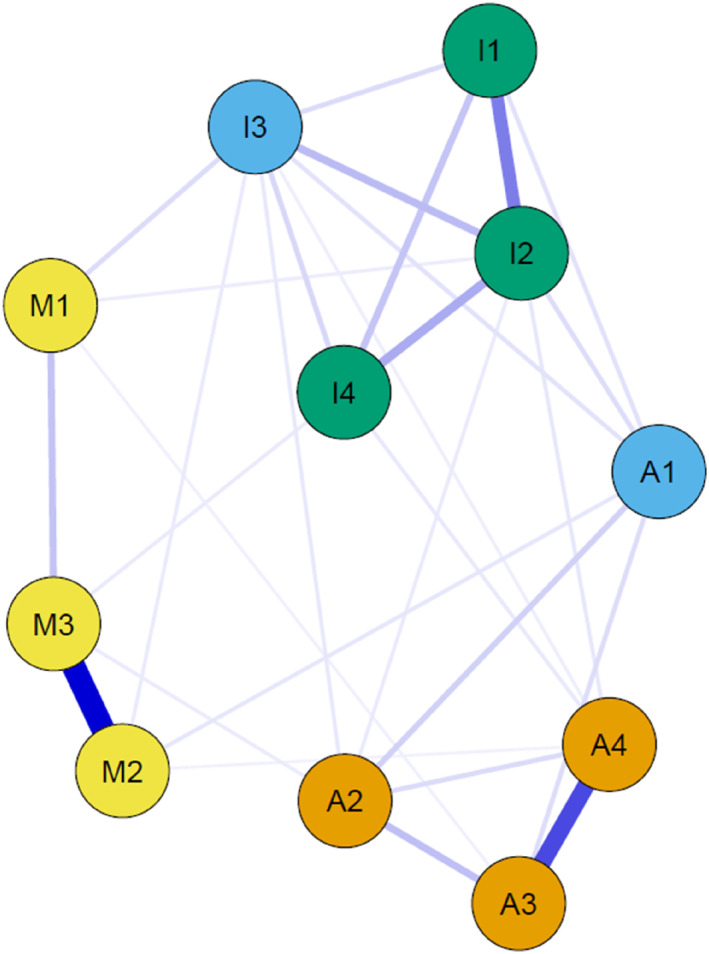
Network of difficult temperament. Blue nodes = bridge nodes; bright yellow nodes = mood nodes; green nodes = intensity nodes; dark yellow nodes = adaptability nodes.

**FIGURE 2:**
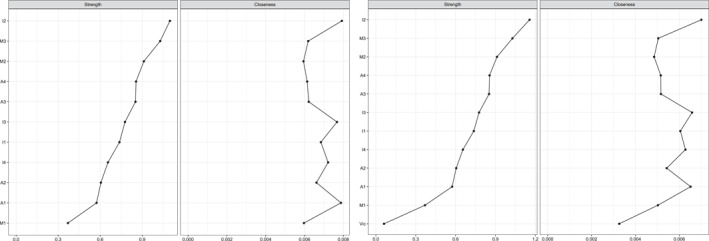
Centrality plot for the network of difficult temperament (left) and network of difficult temperament and peer victimisation (right).

Figure 1 presents the network model for difficult temperament, and Table 2 presents the assigned community, label name, and meaning for each node. The CS‐coefficients indicated that the indices of strength (0.75), closeness (0.52), and BEI (0.67) were stable. Additionally, as shown in Figure S1, the bootstrapped 95% CIs around the edge weights were small, suggesting that the edges did not change significantly across the bootstraps. Using node strength and closeness as measures of centrality (Figure 2; *left*), the most central aspect of difficult temperament to the network was ‘Screams when frustrated’.

**TABLE 2:** Items of the Carey Toddler temperament scale, and their assigned label and community.

Community	Label	Meaning
Mood	M1	Fusses when bottom is wiped after bowel movement
	M2	Unpleasant during face washing
	M3	Unpleasant when hungry and awaiting food
Intensity	I1	Reacts strongly when playing stopped
	I2	Screams when frustrated
	I3	Shows strong reaction to failure
	I4	Shows much body movement when upset
Adaptability	A1	Does not accept delay for desired objects
	A2	Cannot be coaxed from forbidden activity
	A3	Reoffends even if punished firmly
	A4	Goes to areas despite previous warnings

As shown in Figure S1, the strongest edges in the network were formed within each community; however, we also identified bridge nodes that formed several cross‐community edges. The most prominent bridge node was ‘Shows strong reaction to failure’ (*intensity*), which had edges with all other intensity items, as well as edges with mood items (‘Fusses when bottom is wiped after bowel movement’ and ‘Unpleasant when hungry and waiting for food’) and with adaptability items (‘Goes to areas despite previous warnings’, ‘Does not accept delay for desired objects’, and ‘Cannot be coaxed from forbidden activity’). The second most prominent bridge node was ‘Does not accept delay for desired objects’, which had edges with other adaptability items (‘Goes to areas despite previous warnings’ and ‘Cannot be coaxed from forbidden activity’), as well as edges with one mood item (‘Unpleasant when hungry and waiting for food’) and two additional intensity items (‘Screams when frustrated’ and ‘Reacts strongly when playing stopped’).

### Association with victimisation (step 2)

**FIGURE 3:**
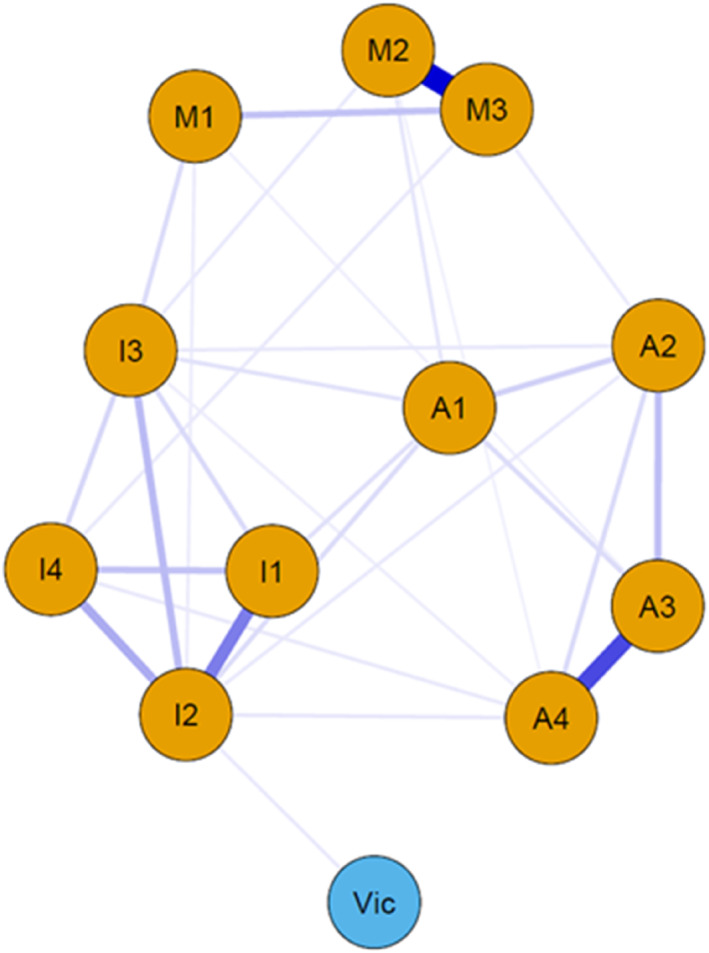
Network of difficult temperament and peer victimisation. Blue node = peer victimisation; dark yellow nodes = difficult temperament nodes.

Figure 3 presents a further network model that includes the node of peer victimisation. As shown in Figure S2, bootstrapped 95% CIs were not large, which suggests that the edges did not vary significantly across the bootstraps. Here, the intensity item ‘Screams when frustrated’ formed an edge with peer victimisation. Similar to the network model in step 1, the most central aspect of this network was ‘Screams when frustrated’ (Figure 2; *right*).

### Moderation by PRS (step 3)

**FIGURE 4:**
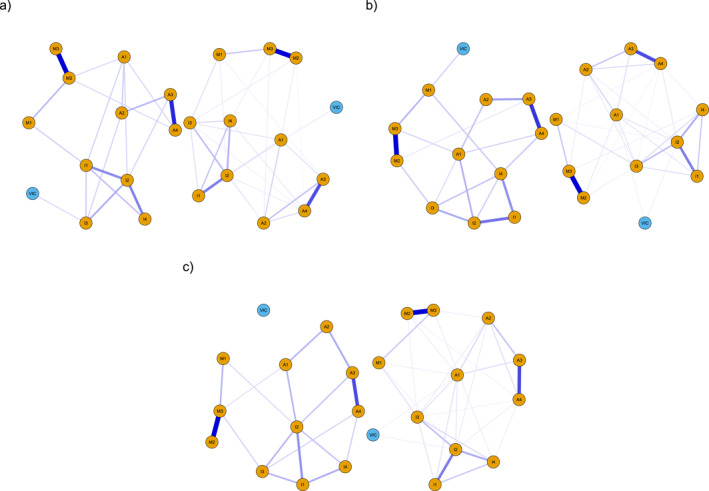
Networks of difficult temperament and peer difficulties, grouped by top 10% (left) versus bottom 90% (right) PRS in ADHD (panel A), schizophrenia (panel B), and depression (panel C). Dark yellow nodes = difficult temperament nodes; VIC = peer victimization. PRS, polygenic risk scores.

Figure 4, panels A, B, and C show the moderated network models that were estimated based on participants in the top 10% and bottom 90% PRS for ADHD, schizophrenia, and depression. Differences were observed in models based on participants at higher polygenic risks (i.e., top 10% PRS). The centrality plots for all 6 networks are included in (Figures S3–S5). For participants high in PRS for neurodevelopmental disorders, aspects of difficult temperament were associated with peer victimisation. For ADHD, ‘Shows strong reaction to failure’ (*intensity*) was associated with peer victimisation. For schizophrenia, ‘Fusses when bottom is wiped after bowel movement’ (*mood*) was associated with peer victimisation. However, difficult temperament nodes did not form edges with victimisation in models based on those with high PRS in depression. To note, these associations were different from the result in step 2, suggesting non‐linear associations between temperamental difficulty and peer victimisation, with respect to genetic risks to mental health problems. Specifically, those with higher genetic propensity for neurodevelopmental disorders, rather than depression, were shown to be especially vulnerable.

On the other hand, network models for participants in the bottom 90% of all three PRS largely resembled the basic model estimated in step 2. For instance, ‘Screams when frustrated’ (intensity) consistently formed an edge with peer victimisation across all models. Additionally, ‘Accepts delay for desired objects’ (adaptability) was also associated with peer victimisation.

When testing differences in structure and global strength, the network comparison test indicated that no differences in structure or global strength were identified between the network models for those with top 10% PRS versus the bottom 90% (Table S2).

### Sensitivity analysis

To examine whether associations between difficult temperament and peer victimisation were different for those with high versus *low* genetic liability for these disorders, we conducted additional network invariance tests using network models estimated with participants in the top versus bottom 10% for each PRS. When comparing those with high versus low PRS for schizophrenia, the network invariance test was marginally significant (*M* = 0.27, *p* = 0.09) and the global strength invariance test was significant (*S* = 0.49, *p* = 0.04). When comparing those with high versus low PRS for ADHD, the network invariance test was nonsignificant (*M* = 0.21, *p* = 0.75), but the global strength invariance test was marginally significant (*S* = 0.42, *p* = 0.08). When comparing those with high versus low PRS for depression, the network invariance test (*M* = 0.22, *p* = 0.56) and the global strength invariance test (*S* = 0.01, *p* = 0.97) were both nonsignificant.

## DISCUSSION

Past research has demonstrated that broad dimensions of early temperamental difficulty act as risk factors for peer victimisation. We built on this research by taking a network approach, examining the network structure of difficult temperament at age 2 and which specific traits are associated with later peer victimisation, as well as testing moderation by polygenic risk. We report three main findings: (i) nodes of temperamental intensity and adaptability had the highest strength, closeness and bridge centrality; (ii) ‘screams when frustrated’ (*intensity*) was associated with peer victimisation; and (iii) aspects of difficult temperament associated with the outcome among those with high ADHD and schizophrenia, but not depression, PRS; additionally, network models for those with typical PRS largely resembled the models from step 2 (i.e., full model with all participants).

### Network model of difficult temperament

The network structure of difficult temperament, consisting of adaptability, intensity, and mood, was first examined using network analysis. We found that nodes central to the network tend to be aspects of difficult temperament associated with emotional and behavioural dysregulation. In particular, the intensity node ‘Screams when frustrated’ formed the most edges with all other nodes in the network at high magnitudes, demonstrating the highest strength and closeness. On the other hand, ‘Shows strong reaction to failure’ and ‘Does not accept delay for desired objects’ were the most prominent bridge nodes, forming the most edges with nodes outside of their communities. This suggests that difficulties in emotional and behavioural self‐regulation, rather than having a predominantly negative mood, could be more central features of difficult temperament as conceptualized by Thomas and Chess (1977). Indeed, a focus on the item‐level associations reveals insights inaccessible to traditional methods, demonstrating the central role of behavioural and emotional regulation challenges in maintaining temperamental difficulties. Interventions targeting these central nodes may therefore be effective in lowering the overall or cross‐domain activation of difficult temperament (Robinaugh et al., 2016).

### Network of difficult temperament and peer victimisation

Partially consistent with the existing literature, we found that ‘Screams when frustrated’ (*intensity*, age 2), an indicator of emotional dysregulation that is also the most central to the difficult temperament network, was associated with peer victimisation (age 8–13; Hanish et al., 2004). Children with difficulties in managing emotional arousal could develop poor social functioning or experience increased distress in potentially threatening peer interactions, which may inhibit effective problem‐solving and prosocial skills to deal with such confrontations, thus putting them at higher risk of peer victimisation (Calkins, 1994; Dodge, 1991; Fogleman et al., 2019; Rosen et al., 2012).

Contrary to past research, however, aspects of difficult temperament characterised by negative mood and behavioural dysregulation did not associate with peer victimisation (Atherton et al., 2017; Peterson et al., 2018). Our results thus suggest that emotional dysregulation (i.e., the *intensity* of emotional expression) is a stronger correlate of peer victimisation than having a predominantly negative *mood* but without intensity in expression. On the other hand, although behavioural dysregulation at a construct level has been found to predict peer victimisation (Atherton et al., 2017; Rosen et al., 2012), no item‐level associations were found. One potential explanation is that poor *adaptability* (e.g., ‘Reoffends even if punished firmly’, ‘Goes to areas despite previous warnings’, and ‘Cannot be coaxed from forbidden activity’) indexes mostly rule‐breaking and deviance, which are facets of behavioural dysregulation less implicated in peer victimisation. In fact, non‐clinical levels of such rule‐breaking behaviours have even been found to associate with higher popularity during adolescence (Sandstrom & Cillessen, 2006).

### Moderation by PRS

In the last step of the analysis, we examined the moderating role of PRS that are associated with peer victimisation and difficult temperament, and found that: (i) difficult temperament was associated with peer victimisation among those with top 10% PRS in ADHD and schizophrenia, (ii) difficult temperament did *not* associate with peer victimisation among those with top 10% PRS for depression, and (iii) network models estimated based on participants with typical (bottom 90%) PRS largely resembled the basic model estimated for all study children.

Regarding the first finding, ‘Show strong reaction to failure’ (intensity) and ‘Fusses when bottom is wiped after bowel movement’ (*mood*) were directly associated with peer victimisation in the network model estimated with participants with high PRS for ADHD and schizophrenia, respectively. Our findings are consistent with the extant literature. For example, PRS for ADHD have been found to associate with temperamental *intensity*, as measured by the CTTS (Riglin et al., 2022) and with emotional dysregulation (Nigg et al., 2020), which could explain why, in this study, peer victimisation was associated with ‘Shows strong reaction to failure’ (*intensity*). Similarly, PRS for schizophrenia have been found to associate with negative *mood*, as measured by the CTTS (Riglin et al., 2022), and more irritability in children aged 7–9 (Riglin et al., 2017). This may be why ‘Fusses when bottom is wiped after bowel movement’ (*mood*), rather than aspects of self‐regulation, was associated with peer victimisation among those with high PRS for schizophrenia.

To note, edge weights identified in the current study tend to be small in effect sizes. However, it is important to consider that a nearly 10‐year time gap exists between difficult temperament and peer victimisation measurements, and the edges identified were partial correlations that remained even after adjusting for the influence of all other nodes in the network. Given that these dimensions of difficult temperament have been found to associate with peer victimisation, the current findings suggest that these nodes could be considered as plausible candidates for causal connections (McNally, 2021).

Regarding the second finding, difficult temperament may have not associated with peer victimisation among those with high depression PRS because traits associated with this outcome emerge later in development. For instance, while PRS for depression and ADHD were both associated with trajectories of irritability, PRS for depression were associated with an adolescent‐onset (i.e., *later*‐onset) trajectory, as opposed to the *early*‐onset persistent trajectory associated with ADHD PRS (Riglin et al., 2019). Similarly, PRS for depression were associated with a *later*‐adolescent onset of depression, while PRS for ADHD and schizophrenia were associated with an *early*‐adolescent onset (Rice et al., 2018). In other words, PRS for depression may be associated with emotional problems with a later onset, while PRSs for neurodevelopmental problems are associated with problems with an earlier onset.

Regarding the third finding, a large overlap (90%) in participants could explain why the network models estimated for those with typical PRS resembled the base model estimated for the overall sample. However, one unexpected result did emerge, where peer victimisation was associated with ‘Does not accept delay for desired objects’ in these network models. Relatedly, results of the network comparisons between the subgroups may have been non‐significant because the test was underpowered. In line with previous findings (van Borkulo et al., 2023), a combination of small sample size and networks of potentially smaller differences may have provided inadequate statistical power. This is supported by our sensitivity analysis, where differences between networks became marginally or statistically significant when comparing the networks from those with high versus low PRS (i.e., more pronounced differences in network). Therefore, the results may be more conservative than the likely true effects in the population, and future studies could examine these item‐level associations and moderation with larger samples.

### Clinical implications

Our findings showed that in a difficult temperament network ‘Screams when frustrated’ (*intensity*) readily activates with other aspects of difficult temperament and was directly associated with peer victimization. This trait may therefore be an important target for intervention, in terms of reducing the overall level of difficult temperament and risks for peer victimisation. Although not directly targeting temperamental difficulty and peer victimization, the Dina Dinosaur Treatment Program has been found to be effective in reducing children's behaviour problems at home and negative peer interactions at school (Webster‐Stratton & Reid, 2003). Strategies of anger management taught to children in the intervention, such as recognizing anger and stopping oneself from acting out, could serve to directly reduce the tendency to ‘Scream when frustrated’, thus reducing overall temperamental difficulty and the risk for peer victimization.

### Limitations and direction for future research

The current findings should be considered in the context of three limitations. First, researchers have raised issues with the failure to replicate network analysis findings (Forbes et al., 2017). However, there is increasing empirical support for the consistency of network structures across time and sample. For example, comparing the networks for symptoms of psychopathology at baseline (T1) and 6‐month follow‐up (T2), Funkhouser et al. (2021) found that 64% of the edges were replicated at T2; moreover, this proportion increased to 84% when comparing networks from T2 and 12‐month follow‐up. Similarly, comparing networks for PTSD across two Dutch and two Danish samples, Fried et al. (2018) found moderate‐to‐high correlations between network structures and centrality estimates. Second, our analytic sample consists of over 98% ethnically White participants; hence, replication of the current findings in more diverse cohorts is necessary to further test their generalisability. Third, disproportionately more at‐risk participants may have been lost to follow up over time. As demonstrated by previous research in ALSPAC (Wolke et al., 2009), this selective attrition could lead to more modest observed estimates than the likely true effects. Fourth, PRS do not typically explain large portions of the phenotypic variance being studied (Murray et al., 2021), which highlights the importance of also evaluating the contributions of, for example, environmental factors in the future. Finally, care should be taken when comparing findings in the neurodevelopmental versus depression PRS, as the source GWAS for these PRS differ in power and quality, with sample sizes varying from 35,374 (ADHD, Demontis et al., 2019) to 807,553 (Depression, Howard et al., 2019), as well as varying levels of heritability of these traits.

### Conclusion

Peer victimisation is a major problem to a substantial proportion of school‐aged children worldwide. We examined the structure of difficult temperament and its associations with later peer victimisation using network analysis, as well as testing the potential moderating role of PRS. Our findings highlight the critical role of emotional and behavioural regulation in the network structure of difficult temperament and in the risk for peer victimisation. Moreover, these associations were found to vary as a function of one's PRS for mental health problems.

## AUTHOR CONTRIBUTIONS

**Tom C.‐H. Wu:** Conceptualization; data curation; formal analysis; visualization; writing—original draft; writing—review and editing. **Deniz Konac:** Methodology; software; visualization; writing—review and editing. **Liliana Garcia‐Mondragon:** Methodology; writing—review and editing. **Alex F. Martin:** Methodology; writing—review and editing. **Barbara Maughan:** Conceptualization; supervision; writing—original draft; writing—review and editing. **Ted Barker:** Conceptualization; funding acquisition; methodology; supervision; writing—original draft; writing—review and editing.

## CONFLICT OF INTEREST STATEMENT

The authors declare no conflicts of interest.

## ETHICAL CONSIDERATIONS

The ALSPAC Ethics and Law Committee and the Local Research Ethics Committees have obtained written informed consent from participants and ethical approval for this study, under proposal B3503 in April 2020.

## Supporting information

Supporting Information S1

## Data Availability

The data that support the findings of this study are available from the ALSPAC study upon request. Restrictions apply to the availability of these data, which were used under licence for this study. The Data are available at the ALSPAC study website through a fully searchable data dictionary and variable search tool (http://www.bristol.ac.uk/alspac/researchers/our‐data/).
