# Supplementary material for: Unpacking early risks for peer victimization: A network analysis of early temperament and polygenic risk scores
Source: JCPP Adv. 2026 Feb 1;6(3):e70092. doi: 10.1002/jcv2.70092 (PMC13339594; doi:10.1002/jcv2.70092)
Supplement: Supplementary file 1 [file JCV2-6-e70092-s001.docx]

**Unpacking early risks for peer victimization: a network analysis of early temperament and polygenic risk scores**

**Supporting Information**

**Appendix S1.** Supplementary description for peer victimisation

Prior to creating factor scores for peer victimisation, competing factor structures have been considered: i) unidimensional model, ii) correlated two-factor model, and iii) bifactor model. We considered the model fit acceptable if the Comparative Fit Index (CFI) and Tucker-Lewis Index (TLI) were above .90 (Bentler & Bonett, 1980), and the root mean square error of approximation (RMSEA) was below .08 (Browne & Cudeck, 1993). The resulting model fit statistics are presented in Table S1. Nested model comparison was then conducted to determine which factor structure was the most appropriate. The results indicated that the bifactor model offered the best fit over the unidimensional (Δχ^2^(9) = 284.94, *p* < .001) and correlated two-factor model (Δχ^2^(8) = 74.70, *p* < .001). Factor analysis was conducted using Mplus version 8.4 (Muthén & Muthén, 2019) and the models were estimated using the robust maximum likelihood estimation.

**Table S1.** Model fit for CFA models

|  | χ² Goodness-of-fit Statistic | CFI | TLI | RMSEA |
| --- | --- | --- | --- | --- |
| Unidimensional model | χ²(27)= 349.22, *p*<.001 | .92 | .89 | .06 |
| Correlated two-factor model | χ²(26)= 119.62, *p*< .001 | .98 | .97 | .03 |
| Bifactor model | χ²(18)= 38.86, *p*< .01 | 1.00 | .99 | .02 |

*Note.* CFI= Comparative fit index, TLI= Tucker-Lewis Index, RMSEA= Root mean square error of approximation

**Figure S1.** Bootstrapped 95% Confidence Intervals of the Edge Weights for Network in Figure 1

**Figure S2.** Bootstrapped 95% Confidence Intervals of the Edge Weights for Network in Figure 2

**Table S2.** Network comparison test coefficients and p-values for global strength invariance (S) and network structure invariance (M)

|  | S (*p-value*) | M (*p-value*) |
| --- | --- | --- |
| ADHD | .01 (.*99*) | .21 (.*12*) |
| Schizophrenia | .17 (*.48*) | .16 (*.71*) |
| Depression | .10 (*.65*) | .18 (*.49*) |

**Figure S3.** Centrality plot for networks of difficult temperament and peer difficulties, grouped by Top 10% (*left*) versus bottom 90% (*right*) PRS in ADHD

*Note*. Z-scores are shown on x-axis

**Figure S4.** Centrality plot for networks of difficult temperament and peer difficulties, grouped by Top 10% (*left*) versus bottom 90% (*right*) PRS in schizophrenia

*Note*. Z-scores are shown on x-axis

**Figure S5.** Centrality plot for networks of difficult temperament and peer difficulties, grouped by Top 10% (*left*) versus bottom 90% (*right*) PRS in depression

*Note*. Z-scores are shown on x-axis
